# Whole-genome sequences of 37 breeding line *Bombyx mori* strains and their phenotypes established since 1960s

**DOI:** 10.1038/s41597-022-01289-3

**Published:** 2022-04-26

**Authors:** Seong-Wan Kim, Min Jee Kim, Seong-Ryul Kim, Jeong Sun Park, Kee-Young Kim, Ki Hwan Kim, Woori Kwak, Iksoo Kim

**Affiliations:** 1 Department of Agricultural Biology, National Academy of Agricultural Science, Rural Development Administration, Wanju, 55365 Republic of Korea; 2 Department of Applied Biology, College of Agriculture & Life Science, Chonnam National University, Gwangju, 61186 Republic of Korea; 3 Gencube Plus, Seoul, 08592 Korea; 4 Hoonygen, Seoul, 08592 Korea

**Keywords:** Animal breeding, Comparative genomics, Genetic engineering

## Abstract

*Bombyx mori* is a key insect in the sericulture industry and one of the very important economic animals that are responsible for not only the livelihood of many farmers internationally but also expended biomedical use. The National Institute of Agricultural Sciences of the Rural Development Administration of Korea (NIAS, RDA, Korea) has been collecting silkworm resources with various phenotypic traits from the 1960s and established breeding lines for using them as genetic resources. And these breeding line strains have been used to develop suitable F1 hybrid strains for specific use. In this study, we report the whole-genome sequences of 37 breeding line *B. mori* strains established over the past 60 years, along with the description of their phenotypic characteristics with photos of developmental stages. In addition, we report the example phenotypic characteristics of the F1-hybrid strain using these breeding line strains. We hope this data will be used as valuable resources to the related research community for studying *B*. mori and similar other insects.

## Abstract (summary)

**Table:** 

Measurement(s)	Larval period • Cocoon yield • Single cocoon weight • Egg color • Cocoon shape • Number of cocoons per liter • Voltinism • Moltinism • Cocoon color• Larva pattern
Technology Type(s)	Clock Device • scale • Eye • amount
Factor Type(s)	Artificial feed • Target Season
Sample Characteristic - Organism	Bombyx mori
Sample Characteristic - Environment	house
Sample Characteristic - Location	South Korea

## Background

The domestic silkworm, *Bombyx mori* (Lepidoptera: Bombycidae), has been domesticated more than 5,000 years ago^1^. It is a key insect in the sericulture industry and one of the very important economic animals that are responsible for the livelihood of many farmers internationally. The sericulture industry, which raises silkworms and obtains silk, is a very labor-intensive primary industry and global production continues to decrease due to a decline of production in China, which accounted for the majority of the world’s raw silk production with India (https://inserco.org/en/statistics). However, it is still one of the most important economic animals and is being used as a new source of income in some developing countries. In addition to the simple use of *B. mori* as silk sources in the textile industry, the use of silkworms and silkworm by-products is further expanded in the fields of drugs, tissue engineering, medical textiles, drug delivery systems, cosmeceuticals, food additives, and manufacturing of valuable biomaterials. Therefore, the importance of *B. mori* as an important animal resource is increasing^2,3^.

As long as the long domestication period of 5000 years, silkworms have been bred to have phenotypes suitable for specific use through strong selection. Domesticated silkworm can produce a large amount of silk and some of them are known to produce 10 times more silk than *Bombyx mandarina*, which is known as a wild type species of *B. mori*^4,5^. However, as the environment of sericulture is changing and the usability of *B. mori* is expanded beyond simple silk production, strains with various phenotypes have the potential to be utilized for various purposes as important biological resources. Because of this importance, even though silk production in general farms is decreasing in South Korea, national research institutes have continuously made efforts to secure useful genetic resources by constructing breeding lines for various strains of *B. mori*. The National Institute of Agricultural Sciences of the Rural Development Administration of Korea (NIAS, RDA, Korea) has been collecting silkworm resources with various expression traits from the 1960s and established a breeding line for using them as genetic resources for F1 hybrid. Strains with various phenotypes can be usefully utilized to enhance specific phenotypes depending on the purpose of use through additional selective breeding and crossbreeding. And they are valuable biological resources to prepare for unexpected environmental changes such as feeding. In addition, the whole-genome sequences of these strains linked to their phenotypes can be used as a major research resource to expand our knowledge of molecular background about *B. mori*.

In this study, we report the whole-genome sequences of 37 breeding line *B. mori* strains established over the past 60 years, along with a description of phenotypic characteristics and photos. These whole-genome sequences linked to the phenotypic characteristics of the established breeding line could be valuable resources for the understanding of *B. mori* genome and provide more insight into the molecular background of various phenotypes.

## Methods

### Construction and maintenance of breeding lines

For the 37 breeding line strains reported in this study, individuals with phenotypic singularities were first produced through two-way or three-way hybridization using locally collected *B. mori strains* after the Korean war. All 37 strains were fixed as a breeding line for F1 Hybrid production through selective self-crossing for a minimum of 10 generations so that the strain could maintain the specific phenotype continuously. The established breeding line strain produces 1 generation per year by hatching and raising eggs from the spring and preserving the eggs secured through self-breeding. Egg incubation is carried out under 16 h of light conditions at 15–26°C and 75–80% humidity. After hatching, 1–3 instars are raised at 25–26°C and humidity of 75–80%, and 4–5 instars are raised at 23-24 degrees and humidity of 65–75%. In all instar stages, mulberry leaves are fed 3 times a day to maintain the breeding line.

### Library construction and data generation

For whole-genome sequencing of 37 breeding line strains, representative male individuals for each strain were randomly selected during the pupa stage. The epidermis tissue was isolated from the pupa and DNA was extracted using the QIAGEN DNesay Blood & Tissue Kit. The extracted DNA was subjected to gel electrophoresis to confirm DNA fragmentation, and trinean, picogreen, bioanalyzer were used to check the quality of the DNA. For five tri-molt mutant strains(KRSM, SH, HS, S7 and SD), the sequencing library was constructed using the MGIEasy DNA Library Prep Kit according to the manufacturer’s protocol and target size of constructed library was 500 bp. 150 bp paired-end data for 5 strains were generated using MGISEQ-2000 sequecing platform. Libraries for remaing 32 strains were constructed using Illumina Truseq Nano DNA LT Kit and target size of constructed library was 700 bp. 150 bp paired-end data for 32 strains were generated using Illumina Nextseq 500.

### Genomics variants and phylogenetic relationship using p50T reference strain

Adapter sequence and low-quality bases were removed by using Trimmomatic^6^ with adapter sequence, and filtered reads were mapped to the reference p50T genome^7^ from NCBI Refseq using bwa-mem2^8^ with default parameter. Removal of PCR duplicated reads and variant calling was performed using samtools^9^, and only biallelic Single Nucleotide Variant(SNV) loci without missing in 38 samples including p50T strain were extracted using VCFtools^10^. InDel and structural variants for each strain were identified using SvABA^11^. All identified variant information can be found in (samtools: https://drive.google.com/file/d/1U3VVh_Q5ER-I6OtcpuqAunHZFtnbaQjG/view?usp = sharing) and (SvABA: https://github.com/asleofn/B_Mori/). Identified SNVs were annotated using SnpEff using custom DB infromation using Refseq annotation. The cladogram was constructed through the Neighbor-joining algorithm using Tassel 5^12^.

## Data Records

The entire data set described in this study is deposited under NCBI Bioproject accession PRJNA751387^13^ and NCBI SRA accession SRP331034^13^ and accession number for each sample can be found in Tables 1 and 2.

**Table 1:** Summary information of generated whole-genome sequence for 37 breeding line *B. mori* strain.

Strain	Intrument	Read Type	Read Count	Length (bp)	Total Bases (bp)	SRA accession
Jam123	Nextseq 500	Paired	62,362,042	151	18,833,336,684	SRR15338622
Jam124	Nextseq 500	Paired	65,995,441	151	19,930,623,182	SRR15338620
Jam125	Nextseq 500	Paired	55,525,443	151	16,768,683,786	SRR15338621
Jam126	Nextseq 500	Paired	61,498,288	151	18,572,482,976	SRR15338615
Jam140	Nextseq 500	Paired	58,794,853	151	17,756,045,606	SRR15338616
Jam143	Nextseq 500	Paired	59,536,298	151	17,979,961,996	SRR15338617
Jam144	Nextseq 500	Paired	60,463,356	151	18,259,933,512	SRR15338618
Jam145	Nextseq 500	Paired	68,058,204	151	20,553,577,608	SRR15338619
Jam149	Nextseq 500	Paired	62,816,706	151	18,970,645,212	SRR15508057
Jam150	Nextseq 500	Paired	58,012,320	151	17,519,720,640	SRR15508056
Jam151	Nextseq 500	Paired	66,515,659	151	20,087,729,018	SRR15508055
Jam152	Nextseq 500	Paired	64,421,250	151	19,455,217,500	SRR15508054
Jam153	Nextseq 500	Paired	64,803,442	151	19,570,639,484	SRR15514279
Jam155	Nextseq 500	Paired	64,822,995	150	19,446,898,500	SRR15514277
Jam156	Nextseq 500	Paired	58,934,629	150	17,680,388,700	SRR15514276
Jam157	Nextseq 500	Paired	59,638,317	150	17,891,495,100	SRR15514275
Jam158	Nextseq 500	Paired	59,925,450	150	17,977,635,000	SRR15514274
Jam159	Nextseq 500	Paired	60,621,724	150	18,186,517,200	SRR15514273
Jam160	Nextseq 500	Paired	73,894,375	150	22,168,312,500	SRR15520445
Jam161	Nextseq 500	Paired	54,746,420	150	16,423,926,000	SRR15520444
Jam162	Nextseq 500	Paired	64,887,891	150	19,466,367,300	SRR15520443
Jam307	Nextseq 500	Paired	59,948,320	150	17,984,496,000	SRR15520442
Jam311	Nextseq 500	Paired	55,223,930	150	16,567,179,000	SRR15520441
Jam312	Nextseq 500	Paired	67,940,967	150	20,382,290,100	SRR15520440
Jam313	Nextseq 500	Paired	63,104,719	150	18,931,415,700	SRR15520439
Jam314	Nextseq 500	Paired	59,084,654	150	17,725,396,200	SRR15521833
Jam315	Nextseq 500	Paired	62,226,371	150	18,667,911,300	SRR15521832
Jam317	Nextseq 500	Paired	55,780,800	150	16,734,240,000	SRR15521830
Jam318	Nextseq 500	Paired	51,913,987	150	15,574,196,100	SRR15521829
Jam319	Nextseq 500	Paired	65,747,515	150	19,724,254,500	SRR15521828
Jam320	Nextseq 500	Paired	57,962,038	150	17,388,611,400	SRR15521827
Jam321	Nextseq 500	Paired	57,346,061	150	17,203,818,300	SRR15521826
KRSM	MGIseq-2000	Paired	199,692,448	150	59,907,734,400	SRR15525308
SH	MGIseq-2000	Paired	57,272,074	150	17,181,622,200	SRR15458431
HS	MGIseq-2000	Paired	52,774,714	150	15,832,414,200	SRR15458432
S7	MGIseq-2000	Paired	59,371,675	150	17,811,502,500	SRR15458433
SD	MGIseq-2000	Paired	49,763,805	150	14,929,141,500	SRR15458430

**Table 2:** Phenotypes, silk production statistics and sequence accession information for 37 *B. mori* breeding lines.

Strain	Larval period (days.hrs)		* Pupation Percentage (%)	** Cocoon yield (Kg)	*** No. of cocoons per liter (EA)	**** Single cocoon weight(g)	***** Cocoon shell percentage (%)	Voltinism	Moltinism	****** Egg color	****** Cocoon color	Cocoon shape	SRA Accession
	5th instar	Total instar											
Jam123	7.2	24.22	86.3	14.9	88	1.77	22.4	2	4	Br	W	Peanut	SRR15338622
Jam124	7.06	25.04	94.7	16.3	66	1.78	22.8	2	4	Br	W	Oval	SRR15338620
Jam125	7.07	26.05	74.7	10.1	92	1.47	20.1	2	4	Br	W	Rectangle	SRR15338621
Jam126	7.06	25.04	93.3	14.8	76	1.85	22.3	2	4	B	W	Oval	SRR15338615
Jam140	7.04	25.02	92.8	15.8	65	1.82	24.6	2	4	Bb	W	Oval	SRR15338616
Jam143	7.08	26.06	80.4	11.1	103	1.51	23.6	2	4	Br	W	Peanut	SRR15338617
Jam144	7.05	25.03	90.5	14.7	62	1.85	22.9	2	4	B	W	Oval	SRR15338618
Jam145	8.04	26.06	95.5	16.1	94	1.69	23.5	2	4	B	W	Rectangle	SRR15338619
Jam149	7.16	25.22	79.9	13	93	1.7	22.4	2	4	Br	W	Rectangle	SRR15508057
Jam150	7	25.22	96	14.9	79	1.65	24.8	2	4	B	W	Oval	SRR15508056
Jam151	7.23	26.21	84.3	14.9	81	1.86	22.5	2	4	Br	W	Oval	SRR15508055
Jam152	8	25.22	91	16.1	58	1.93	21.2	2	4	B	W	Rectangle	SRR15508054
Jam153	7.23	26.21	90.6	16.8	94	1.83	22.3	2	4	B	W	Peanut	SRR15514279
Jam155	6.2	25.02	94.2	17.7	62	2.13	19.2	2	4	Bb	W	Oval	SRR15514277
Jam156	7.16	25.14	94.6	15.6	64	1.95	22.3	2	4	B	W	Rectangle	SRR15514276
Jam157	7.04	26.02	97.9	15.8	96	1.67	21.3	2	4	B	W	Rectangle	SRR15514275
Jam158	8	25.22	93	16.2	61	1.62	28.6	2	4	Br	W	Rectangle	SRR15514274
Jam159	7.04	26.02	94.1	15.4	78	1.75	21.7	2	4	Br	W	Peanut	SRR15514273
Jam160	7.06	25.04	92.3	16.9	73	1.96	22	2	4	Bb	W	Oval	SRR15520445
Jam161	7.22	26.04	86.4	14.2	85	1.87	23	2	4	Br	W	Rectangle	SRR15520444
Jam162	8	25.22	96.8	18.1	61	1.98	23.3	2	4	B	W	Oval	SRR15520443
Jam307	6.21	24.02	82.3	Almost no cocoon (partial sericin cocoon)				2	4	Br	—		SRR15520442
Jam311	6	23.22	96.9	14.5	88	1.58	16.8	2	4	Br	Y	Peanut	SRR15520441
Jam312	7.05	24.21	93	13.3	82	1.65	19.1	2	4	Bb	Y	Oval	SRR15520440
Jam313	5.21	22.02	89	11.2	117	1.35	11.5	2	4	Bb	Y	Rectangle	SRR15520439
Jam314	5.17	20.22	92.9	10	93	1.24	11.7	2	4	Br	Y	Oval	SRR15521833
Jam315	7.16	25.14	91.8	13.4	104	1.63	20.6	2	4	Br	LG	Rectangle	SRR15521832
Jam317	7.16	26.14	87.4	11.6	84	1.55	23.2	2	4	B	W, Y	Oval	SRR15521830
Jam318	8	25.22	91.8	15.7	65	1.69	23.1	2	4	B	W, Y	Oval	SRR15521829
Jam319	6.16	24.14	98.1	16.1	88	1.75	19.3	2	4	B	W, Y	Peanut	SRR15521828
Jam320	6.07	24.05	92	14.5	72	1.78	20.8	2	4	B	W, Y	Rectangle	SRR15521827
Jam321	5.23	23.04	98.5	14.2	96	1.53	15.1	2	4	Br	LG	Rectangle	SRR15521826
KRSM	6.08	25.06	89.1	8.1	144	0.93	10.3	2	3	B	LYG	Oval	SRR15525308
SH	7	24.22	94.9	14.6	96	1.62	18.7	2	3	Br	LO	Oval	SRR15458431
HS	7	23.22	97.7	8.9	164	1.03	12.1	2	3	G	W	Peanut	SRR15458432
S7	6.04	22.02	97.4	8.8	142	1.06	11.6	2	3	B	W	Rectangle	SRR15458433
SD	4.16	22.17	94.9	7.8	152	0.83	10.3	2	3	G	LO	Rectangle	SRR15458430

#Cocoons were produced from 10 thousand of 5 instar larvae.

*Pupation Percentage (%): Probability of pupation from larva.

**Cocoon yield(kg): Weight of 10,000 cocoons containing silkworm pupa.

***No. of cocoons per liter (EA): Number of cocoons in one liter container. (for estimating the size of the cocoon)

****Single cocoon weight (g): Weight of one cocoon.

*****Cocoon shell percentage (%): Ratio of only cocoons to the weight of cocoons containing pupae.

******Color: B, black; W, white; Bb, bright brown; Br, brown; G, gray; Y, yellow; LY, light yellow; LYG, light yellow green; LO, light orange; LG, light green.

## Technical Validation

### Phenotypes and genome sequences of 37 breeding line strains of *B. mori*

**Fig. 1:**
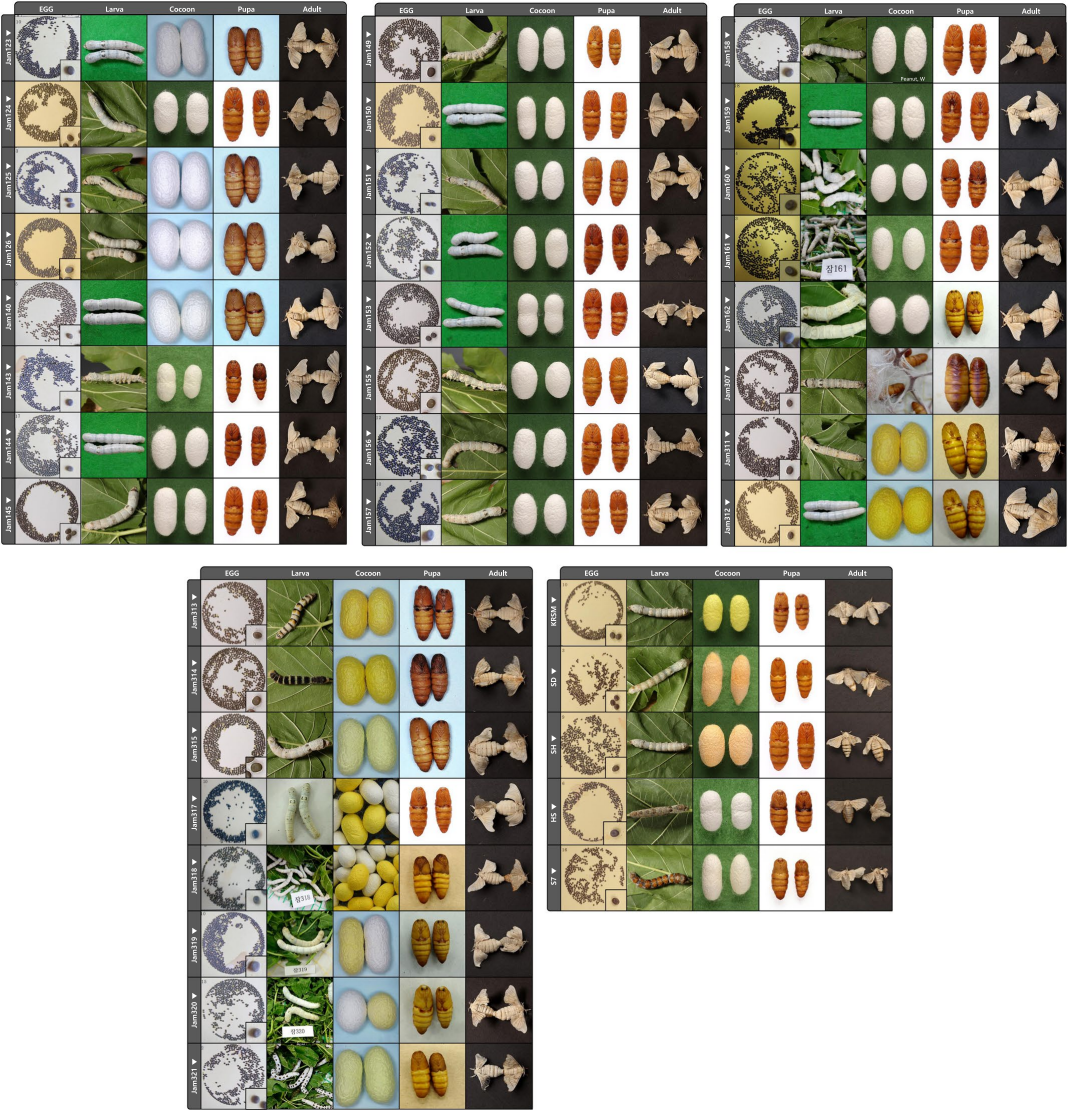
Pictures of egg, larva, cocoon, pupa, and adult of 37 breeding line strains of *B. mori*.

Like other countries where *B. mori* is managed as an important economic animal, the NIAS, RDA, Korea has collected various *B. mori* strains existing in South Korea since the 1960s and established breeding lines of *B. mori* strains as genomic resources. In the early 1970s and 1980s, breeding was carried out cantered on hardy and high silk-producing strains to increase silk production. However, from the 1990s, after Korea’s rapid industrialization, to cope with labor shortages and environmental changes, the focus was on the strains that can use artificial feed, require less labor, and are easily differentiated by gender using larval markings and cocoon colors. The 37 strains reported in this study have important values as seed strains used in the development of customized hybrid strains to respond to changes in the sericulture environment and requests from local farmers. Fig. 1 shows each picture of an egg, larva, cocoon, pupa, and adult from 37 *B. mori* strains. Table 1 shows the summary information of generated whole-genome sequencing data for each strain and Table 2 shows the summary of phenotypic characteristics of 37 breeding line strains with breeding performance. Minimum depth coverage of generated data was over 30X coverage based on the genome size of *B. mori*(about 450 Mb).

**Fig. 2:**
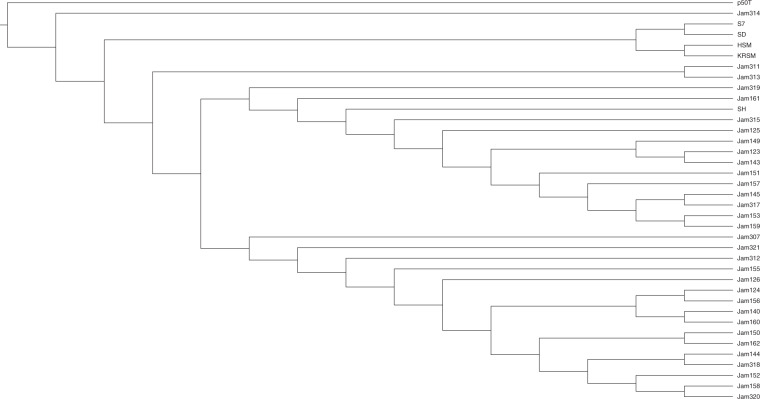
Cladogram of 37 *B. mori* breeding line strains with reference p50T strain using Tassel with Neighbor-Joining method.

Genomic variants for each strain were identified using samtools and SvABA. A total 23,478,741 SNVs were identified from samtools and 1,506,850 SNVs(variant quality under Q30 and multiallelic loci) were filtered. Among 21,971,891 SNVs after filtering, 1,327,196 SNVs located in CDS regions. 1,002,715(75.551%) SNVs were synonymous variants and 324,481(24.449%) SNVs were non-synomymous variants. In InDel and structural variant calling using SvABA performed on individual strains, an average of 622,531 InDels and 41,348 structural variants were identified. All variant calling information is available in the link of method section. To figure out the evolutionary relationship of 37 breeding line strains including P50T, phylogenetic analysis was performed using whole-genome variants from generated sequencing data. Fig. 2 shows the phylogenetic relationship between 37 *B. mori* strains reported in this study with the p50T reference strain. Of the five strains showing tri-molt characteristics, four strains except SH showed a close evolutionary relationship, and some strains had closer evolutionary relationships despite the external differences. Through this, it can be expected that the external characteristics identified by the eye are regulated by the small portion of the total genomics variant and more research will be needed to expand our knowledge for the detailed association between the genomic variants and characteristics. Previously, there were several studies on the phenotype, genetic contents, and regional population of Bombyx mori^14,15^. However, this is the first populatoin-level whole genome data that is released from South Korea, and this is the first data set containing the details of breeding performance and phenotypic characteristics each individual strain. With existing dataset of previous study, more expanded data for understanding the gentic background of silkworm phenotype can be built. And the data reported in this study can be utilized as useful resources for marker development and is expected to help develop silkworm strains with desired traits in a short time through genomic breeding or genetic engineering.

### F1 hybrid strains obtained from 37 breed line strains

**Fig. 3:**
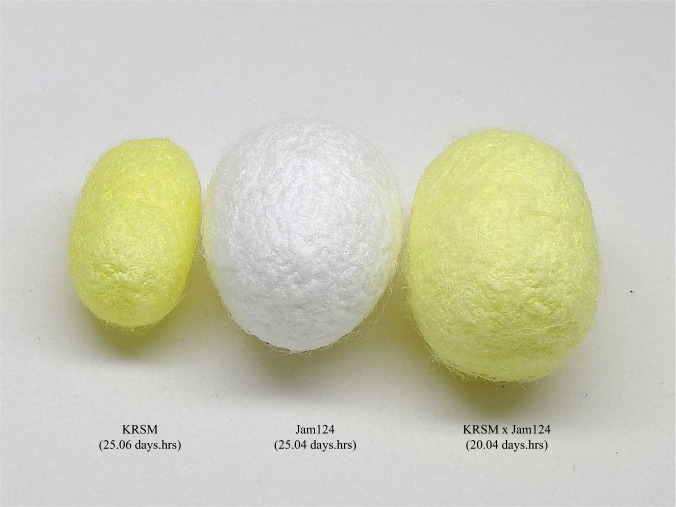
Cocoon of F1 hybrid offspring between male KRSM and female Jam124. All F1 hybrid offspring were tri-molt mutants with a short larval period and the cocoon size was similar to normal *B. mori* with LYG color.

The NIAS, RDA, Korea has produced F1 hybrids with the required phenotypes using the 37 seed strains reported in this study, and generated F1 hybrid strains were annually provided to local farmers. This hybrid strain is selected from several hybrid combinations and they have various characteristics to respond to changes in the breeding environment or purpose of use. Table 3 shows the breeding performance and characteristics of representative F1 hybrid strains constructed using 37 breeding line strains. These strains have several important characteristics and the first of which is whether artificial feed can be used. The silkworm is a monophagous insect whose main diet is mulberry leaves. Mulberry leaves, which are feed for silkworms, require a lot of labor in the process of producing, storing, and providing them. Since sericulture is carried out according to the production time of mulberry leaves, there is a problem that the breeding period is limited throughout the year. If an artificial feed can be fed, the produced mulberry leaves can be utilized more longer and it reduces the labor required to prepare mulberry leaves. And also increased production through year-round feeding can be expected. In addition, they are very important due to the recent rapid climate change. These strains which can be fed artificial feed can flexibly cope with the change in the productivity of mulberry leaves. The second is a sex-limited inheritance strain that can classify gender using larval pattern or cocoon color. In the case of sex classification of silkworms, classification is possible through the tail part of the 5 instar period or the shape of pupa, but if classification is performed using larva’s pattern or color, a lot of labor for gender classification can be effectively reduced. The third is a hybrid strain that produces color silk. Among the 37 breeding line strains, the strain producing cocoons with yellow and light green colors has a lower cocoon size compared to the general strain for silk production. Therefore, hybrid strain is a strain that effectively improved the existing low color silk production. In addition to the direct use of color silk itself, these strains can be used as functional strains for carotenoids or flavonoids required for color silk generation. The fourth is a strain that does not produce a cocoon. The breeding line strain Jam307 in this study produces very few cocoons. Only about 1.2% of individuals produce fibroin-free, sericin-only nets. By dissecting the silk gland of this strain, it can be seen that the posterior silk gland, which is important for fibroin-based filamentation, is degenerated. In the Jam307 x Jam126 hybrid strain, which produces relatively large larva and pupa compared to Jam307, most individuals form sericin nets and normal silk with fibroin was not generated. Through this, it can be expected that the characteristic of Jam307, which produces silk composed only of sericin due to the degeneration of the posterior silk gland, is a dominant trait. This hybrid strain that does not make a cocoon is mainly utilized to use the silkworm itself, such as cordyceps production and silkworm powder for a food additive. Lastly, the most recently developed strain is a hybrid strain of KRSM and Jam124. The phenotypic results were not included in Table 3 because the breeding performance evaluation was not completed yet, but the KRSM x Jam124 hybrid strain has the following characteristics. The KRSM x Jam124 hybrid strain produces light green silk like tri-molt characteristics like *B. mori* KRSM, but the silk production is similar to the general silk production strain. Fig. 3 shows the cocoons of KRSM, Jam124, and KRSM x Jam124 hybrid strains. The cocoon size of the hybrid strain is almost similar to the silk production strain Jam124. In addition to the increased cocoon size, the total larval period was surprisingly shortened. Unlike KRSM and Jam124, which have larval periods of 25.06 and 25.04 days.hrs, respectively, the total larval period of this hybrid strain was 20.04 days.hrs. It is about 20% shorter than the original strains. Since a 20% reduction in production time can increase silk production as well as reduce the production cost, the hybrid strain is being developed as a useful resource that can contribute to productivity improvement. In addition, the whole genome sequences reported in this study can help to provide more insight into the genetic background of *B. mori* phenotype and develop modified strain for specific use using genetic engineering.

**Table 3:** Summary of phenotypic characteristics and breeding performance of F1 hybrid strains.

Hybrid Strain	Larval Period (days.hrs)	* Pupation Percentage (%)	** Cocoon yield (Kg)	*** No. of cocoons per liter (EA)	**** Single cocoon weight(g)	***** Cocoon shell percentage (%)	Moltinism	Cocoon color	Cocoon shape	Target Season	Characteristics
Jam123 x Jam124	24.02	95.4	24.2	61	2.56	24.9	4	W	Rectangle	Spring, Fall	^※^Artificial Feed
Jam125 x Jam126	22.19	94.3	22.3	62	2.41	24.4	4	W	Rectangle	Fall	^※^Artificial Feed Sex determinant using larva pattern (♂:X, ♀:O)
Jam125 x Jam140	23.18	95.8	23.6	53	2.48	25.2	4	W	Rectangle	Spring, Fall	^※^Artificial Feed
Jam143 x Jam144	23.13	95.9	20.8	54	2.25	24	4	W	Rectangle	Spring, Fall	Sex determinant using larva pattern (♂:X, ♀:O)
Jam307 x Jam126	23.06	82.8	Sericin cocoon			—	4	—	—	Fall	Sericin cocoon
Jam151 x Jam152	24.15	95.7	25	48	2.71	24	4	W	Rectangle	Spring	Sex determinant using larva pattern (♂:X, ♀:O)
Jam311 x Jam312	22.23	95.3	18.9	67	1.99	19.7	4	Y	Rectangle	Spring	Yellow Silk Production
Jam315 x Jam316	23.01	96.7	21.9	56	2.31	23.1	4	LG	Rectangle	Spring, Fall	Light Green Silk Production
Jam153 x Jam154	25.04	94.1	21.4	56	2.32	24.7	4	W	Rectangle	Spring	Sex determinant using larva pattern (♂:X, ♀:O)
Jam155 x Jam156	24.15	96.4	25.4	44	2.72	23.2	4	W	Rectangle	Spring	Big Larva Size, High Silk Production
Jam157 x Jam158	23.18	95.9	22.6	57	2.4	23.6	4	W	Rectangle	Spring	^※^Artificial Feed
Jam317 x Jam318	24.06	93.2	21.3	54	2.29	24	4	W, Y	Rectangle	Spring	Sex determinant using cocoon color (♂:W, ♀:Y)
Jam161 x Jam162	22.04	92	18.2	51	2.08	23.5	4	W	Rectangle	Spring, Fall	Sex determinant using larva pattern (♂:X, ♀:O)
Jam319 x Jam320	24.03	94.9	20.6	60	2.22	21.8	4	W,Y	Rectangle	Spring, Fall	Sex determinant using larva pattern and cocoon color (♂:X, W, ♀:O, Y)

#Cocoons were produced from 10 thousand of 5 instar larvae.

*Pupation Percentage (%): Probability of pupation from larva.

**Cocoon yield(kg): Weight of 10,000 cocoons containing silkworm pupa.

***No. of cocoons per liter (EA): Number of cocoons in one liter container. (for estimating the size of the cocoon).

****Single cocoon weight (g): Weight of one cocoon.

*****Cocoon shell percentage (%): Ratio of only cocoons to the weight of cocoons containing pupae.

******W, white; Y, yellow; LG, light green.

※Artificial Feed: Pupation possible only using artificial feed in all stage.

## Data Availability

All generated sequencing raw reads have been deposited in the NCBI Sequence Read Archive under accession PRJNA751387. The following commands were used to identify the phylogenetic relationship between breeding line strains. <Adapter Trimming: Trimmomatic v0.39> java -jar trimmomatic-0.39.jar PE -threads 12 ILLUMINACLIP:<Adapter Fasta>:2:30:10:2:keepBothReads LEADING:3 TRAILING:20 MINLEN:125<Read Mapping: bwa-mem2 v2.1> bwa-mem2 mem -t 16 <reference_index> <sample_left_pair> <sample_right_pair> | samtools sort –o <sample_name>.bam – <Remove Duplicate: samtools v1.10> samtools rmdup <aligned_bam_file> <Remove_duplicated_bam_file> <Variant Calling: bcftools v1.10.2> bcftools mpileup -Ou –f <reference_file> -s <bam_list_file> | bcftools call -mv -Ov -o calls.vcf <Variant Filtering: Vcftools v0.1.16> vcftools --vcf calls.vcf --remove-indels --recode --max-missing 1.0--min-alleles 2 --max-alleles 2 --minQ 30 <InDel and SV calling: SvABA v1.1.3> svaba run –t <bam_file> -p 12 -L 6 -I –a <sample_name> -G GCF_014905235.1_Bmori_2016v1.0_genomic.fna <SNV annotation: SnpEff v5.0> Java -jar snpEff.jar Mori <SNV.vcf>
